# Effect of Wet Fractionation Conditions and Pulsed Electric Field on Arabinoxylan and Protein Recovery from Maize

**DOI:** 10.3390/foods14050760

**Published:** 2025-02-23

**Authors:** Ulrich Sukop, Katharina Hoefler, Denisse Bender, Stefano D’Amico, Mario Jekle, Regine Schoenlechner, Konrad J. Domig

**Affiliations:** 1 Department of Biotechnology and Food Science, Institute of Food Science, BOKU University, Muthgasse 18, 1190 Vienna, Austria; ulrich.sukop@boku.ac.at (U.S.); konrad.domig@boku.ac.at (K.J.D.); 2 Department for Feed Analysis and Quality Testing, Institute for Animal Nutrition and Feed, AGES–Austrian Agency for Health and Food Safety, Spargelfeldstraße 191, 1220 Vienna, Austria; katharina.hoefler@ages.at (K.H.); stefano.d-amico@ages.at (S.D.); 3 Department of Biotechnology and Food Science, Institute of Food Technology, BOKU University, Muthgasse 18, 1190 Vienna, Austria; regine.schoenlechner@boku.ac.at; 4 Department of Plant-based Foods, University of Hohenheim, Stuttgart, Garbenstraße 25, 70599 Stuttgart, Germany; mario.jekle@uni-hohenheim.de

**Keywords:** corn, cereal processing, steeping, hemicellulose, functional properties, wet-milling

## Abstract

Maize wet fractionation by-products are primarily used as feed but offer potential for food applications. Arabinoxylans (AXs) and proteins are particularly valuable due to their network-forming properties, which depend on their molecular structure. This study assessed the effect of the steeping conditions (acid type and pH variation) combined with a pulsed electric field (PEF) as a strategy for recovering these polymers, while also evaluating their effect on the recovery yield, fraction composition, and key AX characteristics. The physical properties were studied in selected fractions to investigate the process-induced structural changes. Lactic acid and hydrochloric acid (pH 2.5) were most effective in enhancing AX and protein recovery in fiber-rich (FF) and protein-rich (PF) fractions, respectively, while acetic acid exhibited the lowest efficiency. However, bound polyphenols were best retained in the FF when lactic acid was used, indicating the lowest structural damage to AXs, compared to other acids and using a higher pH. Additional PEF pre-treatment significantly enhanced the release of proteins, dietary fiber, and fat from the FF while inducing physical modifications to the fractions (PF: higher protein unfolding, FF: improved water-binding, pasting when using PEF). These findings highlight the potential of optimizing the processing conditions to adjust the recovery of proteins and AXs from maize, while minimally affecting their functionality.

## 1. Introduction

Wet fractionation is commonly used for processing maize to produce relatively pure fractions of starch, germ, bran and proteins. While starch is the primary product of interest, the remaining fractions are often undervalued and used as animal feed or discarded as waste, despite their exceptional nutritional and functional properties [1,2,3].

Among them, cereal brans can serve as rich sources for the extraction of hemicellulosic fractions, such as highly branched and feruloylated arabinoxylans (AXs). These bioactive compounds show several remarkable functional properties, including emulsification, thickening, film-forming and gelling [1]. Maize AXs, in particular, exhibit an excellent crosslinking potential, enabling the formation of a strong and heat-resistant gel network through covalent ferulic acid (FA) bonds [4]. This crosslinking potential has led to their application as structuring agents in several food products, including gluten-free (GF) bread [5]. Similarly, maize proteins have gained attention as promising gluten substitutes due to their network-forming properties [6]. When applied together, proteins and AXs can crosslink with each other, forming complex networks that influence the viscoelasticity of various food matrices [5,7]. However, to induce such a protein–AX network, both polymers must possess a suitable molecular structure as well as a sufficient number of accessible ferulic acid and tyrosine units, respectively [8], making their recovery or extraction challenging.

Wet fractionation may be used as a first step to effectively separate and enrich AXs and proteins from the grain. However, the impact of such processing on the structural integrity of these polymers remains unexplored. Industrial wet fractionation of maize has primarily focused on maximizing the recovery and purity of starch, often neglecting the effects of processing on the dietary fiber and protein in the by-products. Developing an optimized fractionation process that achieves efficient separation while preserving key structural properties of AXs and proteins is therefore important.

The separation efficiency of a wet fractionation process strongly depends on the initial steeping phase [9], which softens the grain and enhances the protein solubilization. The key factors influencing steeping mainly include the acidic conditions, such as the pH and the type of organic acid, but also the presence of sulfur dioxide and the steeping temperature and time [10]. Tailoring the steeping conditions is essential to balance effective nutrient recovery with minimal degradation of the polymer properties.

In recent years, pulsed electric field (PEF) technology has emerged as a gentle, non-thermal processing method in the food sector, which facilitates the extraction of target compounds [11]. Beyond its extraction potential, PEF may induce structural modifications in food matrices, affecting polymers such as proteins and dietary fibers [12,13]. Given these effects, integrating PEF as a pre-treatment step in traditional wet fractionation processes may offer an innovative strategy to enhance the separation of our target components; however, its impact on the resulting fractions after processing needs to be evaluated.

Based on the current knowledge gaps, this study aimed to evaluate the effect of wet fractionation on the recovery yield and key structural parameters of AXs and proteins in maize fractions. A key focus was placed on varying the steeping conditions (lactic, acetic, hydrochloric acid; pH 2.5 and 3.5), which were identified as critical separation parameters. These conditions were analyzed for their influence on the fractionation yield and key structural properties of AXs, such as the branching degree and AX-bound phenolic acids, which are relevant for polymer functionality (i.e., crosslinking). Moreover, this study aimed to provide the first insights into the potential of PEF as an approach to further enhance the separation efficiency of a wet fractionation process, while also assessing the process-induced modifications within the recovered fractions.

## 2. Materials and Methods

### 2.1. Materials

The maize variety DKC3972 was kindly donated by Biohof Schmidt (Neudorf bei Staatz, Austria). All the chemicals were of analytical grade and purchased from Carl Roth GmbH + Co. KG (Karlsruhe, Germany) and Sigma-Aldrich (Steinheim, Germany).

### 2.2. Lab-Scale Wet Fractionation: Experimental Setup

**Figure 1:**
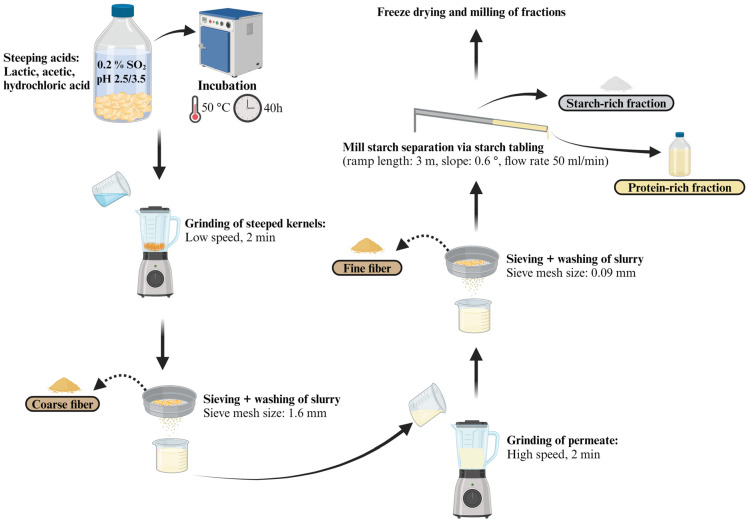
Lab-scale wet fractionation process of maize yielding three different fractions: fiber-rich fraction (combined coarse and fine fiber), protein-rich fraction and starch-rich fraction (created with BioRender.com).

The lab-scale fractionation process (Figure 1) was conducted according to Eckhoff et al. [14] and Yang et al. [15] with modifications. The pH conditions of 2.5 and 3.5 were selected based on the separation yield reported in these studies. Initially, the impact of steeping at pH 2.5, which was adjusted with lactic, glacial acetic, or hydrochloric acid, was tested. The acid yielding the highest protein and dietary fiber recovery was selected for the trials at pH 3.5.

#### 2.2.1. Steeping

For steeping, 400 g of kernels were added to 1200 g of a 0.2% (*w*/*w*) sulfur dioxide solution (using 3.56 g sodium metabisulfite) with lactic acid, acetic acid or hydrochloric acid (pH value of 2.5/3.5). The steeping process was conducted for 40 h at 50 °C in a drying cabinet (TIN-TF50, Phoenix Instrument GmbH, Garbsen, Germany). Afterwards, the kernels were separated from the steeping water and transferred to a blender (Cooking Blender HR2088/91, Philips, Amsterdam, The Netherlands).

#### 2.2.2. Fiber Recovery

The soaked maize kernels were ground with distilled water (1:1 ratio) at low speed for 2 min. The slurry was sieved through a 1600 µm mesh (Haver & Boecker OHG, Germany) to separate the coarse fiber fraction, which was washed with 300 mL of water and set aside. The filtrate was returned to the blender for fine fiber separation. A second high-speed grinding (step 9, 2 min) was followed by sieving through a 90 µm sieve (Haver & Boecker OHG, Oelde, Germany) and washing with 300 mL distilled water. The fine and coarse fibers were combined, and the filtrate (mill starch) was separated.

#### 2.2.3. Starch–Protein Separation

The starch was separated by pumping the mill starch (flow rate = 50 mL/min) onto an inclined sedimentation table (3 m × 0.75 m; 0.0104 cm/cm gradient (=0.6°)). The protein-rich solution was collected at the table’s end, followed by washing the starch sediment with 300 mL distilled water to enhance protein recovery. The starch-rich fraction (SF) was dried at room temperature for one hour before manual removal.

### 2.3. Pilot-Scale Wet Fractionation

Upscaling of the best lab-scale fractionation conditions involved minor modifications: 12.5 kg of maize kernels were steeped in 37.5 kg of steeping solution (0.2% SO_2_; lactic acid, pH 2.5) for 40 h. The steeped kernels were coarsely ground with distilled water (1:1) using a GKM Alexanderwerk cutter (Remscheid, Germany), followed by milling (cutting ring gap size: 0.5 mm) using a STEPHAN Microcut wet mill (Hameln, Germany). The slurry was divided into two portions: one for the PEF pre-treatment (see Section 2.4) and the other portion (untreated) to remove coarse fibers using a custom-made sieve (length: 1 m, width: 0.5 m; mesh size: 1 mm). After washing with 1 L water, a second milling step (cutting ring gap size: 0.05 mm) and sieving (custom-made sieve mesh size: 100 µm) were performed. The coarse and fine fibers were combined and the mill starch was processed using a sedimentation table (see Section 2.2.3).

### 2.4. Pre-Treatment with Pulsed Electric Field (PEF)

PEF treatment of the maize slurry was conducted using a pilot-scale batch system (ScandiNova Systems AB, Uppsala, Sweden), including a treatment chamber with stainless steel plate electrodes (distance of 9 cm). Before treatment, the electrical conductivity and temperature of the slurry were measured using a digital thermometer (Testo 922, Testo GmbH, Vienna, Austria) and a conductivity meter (FiveGo F3, Mettler Toledo, Greifensee, Switzerland). The PEF processing parameters included the following: rectangular monopolar-shaped pulses; pulse width, 5 µs; field strength, 1.5 kV/cm; pulse number, 4718; pulse frequency, 100 Hz; current, 202 A; voltage set, 16.8 kV; voltage real, 17 kV; total specific energy input, 50 kJ/kg. The PEF process was carried out in 10 batches of 1620 g each (below 25 °C). The processed maize slurry was further fractionated as in Section 2.3.

### 2.5. Sample Preparation

All the lab-scale and pilot-scale fractions were frozen at −40 °C and freeze-dried (ZIRBUS technology GmbH, Bad Grund, Germany) at 0.5 mPa for 72 h. The maize fractions and kernels were milled using a Retsch Centrifugal Mill ZM 200 (Haan, Germany) with a 0.5 mm sieve for nutritional and functional characterization.

### 2.6. Fraction Yield and Nutrient Recovery Yield

The calculation of the fraction yields was based on the ratio of the dry weight of the fraction to the total dry weight of the maize kernels according to Equation (1):

(1) $$\mathrm{Fraction}\;\mathrm{yield}\;[\%]=\frac{\mathrm{Dry}\;\mathrm{weight}\;\mathrm{of}\;\mathrm{fraction}\;[g]\;\times \;100}{\mathrm{Dry}\;\mathrm{weight}\;\mathrm{of}\;\mathrm{kernels}\;[g]}$$

The nutrient recovery yield was defined as the quantity of a given nutrient that was recovered from the whole kernel within a fraction (based on dry matter), calculated according to Equation (2):

(2) $$\mathrm{Nutrient}\;\mathrm{recovery}\;\mathrm{yield}\;[\%]=\frac{\mathrm{Nutrient}\;\mathrm{content}\;\mathrm{in}\;\mathrm{fraction}\;[g]\;\times \;100}{\mathrm{Nutrient}\;\mathrm{content}\;\mathrm{in}\;\mathrm{whole}\;\mathrm{meal}\;\mathrm{flour}\;[g]}$$

### 2.7. Chemical Characterization of Maize Fractions

**Figure A1:**
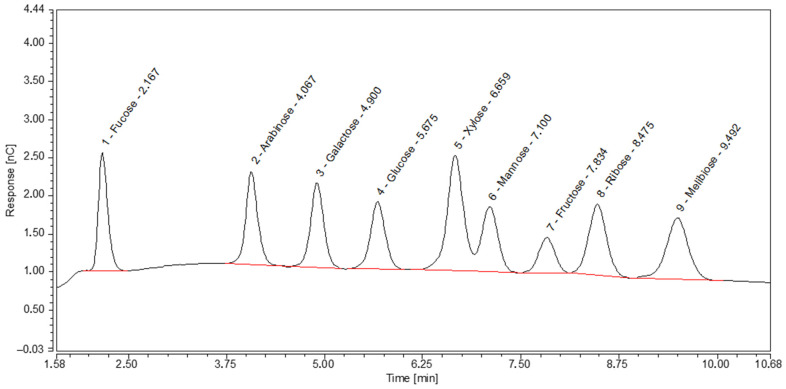
Chromatogram of the HPAEC-PAD calibration standard separation at a level of 0.1 mg/L with the corresponding retention times at a flow rate of 0.2 mL/min and a PA-20 Fast Column tempered at 30 °C.

The chemical composition of the wholemeal flour and fractions was assessed by the following standard methods. The dry matter was determined according to AACC standard method 44-19 [16]. The total starch content was measured using a Total Starch Assay kit (Megazyme International Ireland Ltd., K-TSTA-100A, Wicklow, Ireland) based on the AACC method 76-13.01 [16]. The total dietary fiber (TDF) content was determined according to a total dietary fiber assay procedure (Megazyme International Ireland Ltd., K-TDFR-200A, Wicklow, Ireland) following the standard method of AACC No. 32-07.01 [16]. Additionally, the total nitrogen content was determined according to the Kjeldahl method of the ICC standard No. 105/2 [17]. The fat content was determined based on the Soxhlet method of ICC standard No. 136b [17]. The ash content was measured in accordance with the AACC method No. 08-01 [16]. The measurement of the total phenolic acids content was carried out following the procedure of Speranza et al. [18] with slight adaptions to the sample weight (0.5 g) and volumes of reagents during the bound phenolic acid extraction procedure: 3 mL of 80% ethanol, 2.1 mL of 2 M sodium hydroxide, 450 µL 12 M hydrochloric acid, 1.4 mL ethyl acetate. The AX content was determined via acid hydrolysis and high-performance anion-exchange chromatography with pulsed amperometric detection (HPAEC-PAD) [19]. Samples (200 ± 10 mg) were suspended in 5 mL 1.9 M trifluoroacetic acid (TFA), vortexed for 1 min and hydrolyzed at 103 °C for 2.4 h (Durocell Eco line oven, MMM Group, Munich, Germany) with shaking of the samples every 30 min. After cooling with 50 mL cold ultra-high-quality (UHQ) water, the non-hydrolyzed macromolecules were precipitated by adding 50 µL Carrez I (15% potassium hexacyanoferrate solution) and II (30% zinc sulfate solution), followed by centrifugation (10 min, 2000× *g*, 20 °C). The supernatant aliquots (0.5 mL) were vacuum-dried (45 °C, 3 h, <0.013 bar) (Savant SpeedVac SPD1030, Thermo Fisher Scientific, Sunnyvale, CA, USA), resuspended in 1 mL UHQ water, vortexed (1 min), and filtered (0.25 µm) and diluted accordingly to the calibrated range of 0.1–20 mg/L. HPAEC-PAD analysis was performed using a Dionex^TM^ ICS-6000 DC system (Thermo Fisher Scientific, Sunnyvale, CA, USA) with a CarboPac^TM^ PA20 Fast (2 × 100 mm, 4 µm) and guard column at 30 °C, a gradient elution (with eluent B = 200 mM NaOH and C = 1 M NaOH at 0–7.5 min 1% B; at 7.5–12.5 min 7% B; at 12.5–22.5 min 17% B; at 22.5–32.5 min 100% B; at 32.5–32.6 min 0% B and 20% C; at 32.6–37.5 min 30% B and 70% C; at 37.5–40 min 30% B and 70% C; at 40–40.1 min 1% B and 0% C and at 40.1–55.0 min 1% B and 0% C) and a 0.2 mL/min flow rate. Quantification was carried out using an external calibration calculated as a quadratic regression with an R^2^ of >0.99 at five calibration points (0.1 mg/L, 0.5 mg/L, 1 mg/L, 5 mg/L, 10 mg/L, 20 mg/L), including fucose, arabinose, galactose, glucose, xylose, mannose and fructose. The separation of the calibration standards, including their retention times, is shown in Appendix Figure A1. The AX content was calculated as the sum of arabinose and xylose. All the chemical analyses were carried out in triplicate.

### 2.8. Analysis of Functional Properties

All the analyses were performed in triplicate and the results were expressed as a proportion of the total dry weight of the measured sample.

#### 2.8.1. Determination of Pasting Properties Using RVA

The pasting properties of selected pilot-scale fractions were determined using a Rapid Visco Analyzer (RVA-4500, PerkinElmer, Waltham, MA, USA) and Thermocline for Windows v2.2 (Newport Scientific Pty. Warriewood, NSW, Australia) following the ICC standard method No. 162 [17].

#### 2.8.2. Solvent Retention Capacity (SRC) Profile

The SRC analysis of the wholemeal maize flour and maize fiber-rich fraction and PF were performed according to AACC No. 56-11 [16] with slight adaptions. Samples (5 g) were mixed with 25 mL of the respective solvent used in the SRC method, either deionized water or a 50% (*w*/*w*) sucrose solution, and shaken vigorously. The suspension was allowed to swell for 20 min at 20 °C, with shaking every five minutes. Samples were then centrifuged (1029× *g*, 15 min), decanted and allowed to drain for 10 min. The pellet (gel) was weighed and the SRC was calculated using Equation (3).

(3) $$\mathrm{SRC}\;[\%]=\left[\frac{\mathrm{Gel}\;\mathrm{weight}\;[g]}{\mathrm{Sample}\;\mathrm{weight}\;[g]}\times \left(\frac{86}{100-\mathrm{Moisture}\;\mathrm{content}\;\mathrm{of}\;\mathrm{sample}\;[\%]}\right)-1\right]\times 100$$

#### 2.8.3. Protein Surface Hydrophobicity

The protein surface hydrophobicity was measured according to Waziiroh et al. [20]. Approximately 100 mg of each pilot-scale PF, as well as wholemeal flour, was dissolved in 10 mL of a 20 mM phosphate buffer (pH 5.5; indicating a gluten-free batter). A 1 mL aliquot was mixed with 200 µL of a 0.1% bromophenol blue (BPB) solution. A control with only 200 mg of BPB solution and 1 mL phosphate buffer was also prepared. The samples were shaken for 10 min (20 °C) and then centrifuged at 2000× *g* for 15 min. The absorbance (A) was measured at 595 nm against the blank (phosphate buffer). If the absorbance exceeded the spectrophotometer’s linear range (0.100–1.20 nm), the supernatant was diluted with phosphate buffer. The BPB binding was calculated according to Equation (4):

(4) $$\mathrm{BPB},\;\mathrm{bound}\;[µL]=200\;µL\;\times \frac{\left(A_{\mathrm{control}}-A_{\mathrm{sample}}\right)}{A_{\mathrm{control}}}\;\times \;\mathrm{dilution}\;\mathrm{factor}$$

#### 2.8.4. Protein Solubility

The protein solubility was determined following International Organization for Standardization (ISO) 14244:2014 [21] with slight modifications. Approximately 1.5 g of the pilot-scale PF and wholemeal flour were each placed into two separate 50 mL falcon tubes, both containing 37.5 mL of distilled water. The samples were stirred for 20 min, then centrifuged (10 min, 800× *g*). Then, 7.5 mL of the supernatants from both tubes were transferred into a Kjeldahl tube (15 mL in total) and incubated at 70 °C overnight. The total nitrogen content according to the Kjeldahl method of ICC standard No. 105/2 [17] was determined. The protein solubility was calculated according to Equation (5). The aliquot corresponded to a factor of 5 (15 mL of supernatant from a total of 75 mL)

(5) $$\mathrm{Protein}\;\mathrm{solubility}\;[\%]=\frac{\mathrm{Soluble}\;\mathrm{protein}\;\left[\%\right]\times 100\times \mathrm{Aliquot}}{\mathrm{Crude}\;\mathrm{protein}\;[\%]}$$

### 2.9. Statistical Data Analysis

The results were statistically analyzed using the software Statgraphics Centurion 19–X64 (Statpoint Technologies, Warrenton, VA, USA) and expressed as the mean ± standard deviation. A one-way analysis of variance (ANOVA) and Fisher’s least significance tests were used to estimate the significant differences between samples (*p*-value < 0.05).

## 3. Results and Discussion

### 3.1. Effect of Steeping Acids and pH on Maize Fraction Properties and Yield (Lab-Scale)

**Figure 2:**
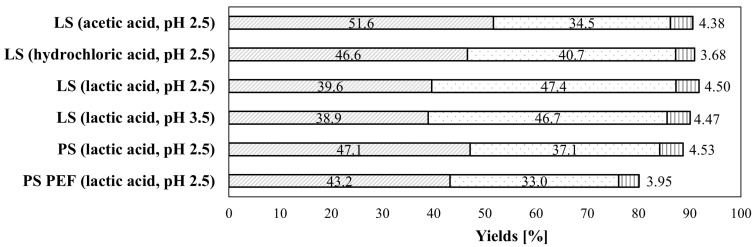
Fraction yields [% of wholemeal flour], based on dry matter, of lab-scale (LS) as well as pulsed electric field (PEF) pre-treated and untreated pilot-scale (PS) maize fractions using different steeping acids and pH values.

Table 1 displays the effect of the steeping acids and pH on the nutritional distribution of lab-scale fractionated maize compared to wholemeal maize flour. Figure 2 and Table A1 show the yields of each fraction and nutrient recovered from the whole maize kernels, respectively. The results revealed that the wet fractionation process successfully yielded three distinct fractions (fiber-rich (FF), starch-rich (SF), protein-rich (PF) fractions). Furthermore, the fractionation process achieved a total solid recovery of 90–91%, with only low handling losses in-between processing steps (see Figure 2). It is noteworthy to mention that the maize was not degermed prior to fractionation to enhance the protein recovery. This led to an uneven distribution of the germ among the aforementioned fractions, especially in the FF and PF, and affected both the yield and nutritional composition of the fractions. Steeping with acetic and hydrochloric acid (pH 2.5) resulted in a higher FF yield and a lower recovery of the SF compared to steeping with lactic acid at either pH (2.5 or 3.5), as seen in Figure 2. The PF yield was similar in all the lab-scale fractionations and ranged from 3.7% to 4.5%. Yang et al. [15] confirmed the significant impact of various steeping acids and SO_2_ sources on the wet-milling fraction yields and starch properties of two maize hybrids. These findings revealed that lactic acid and, contrary to the present study, acetic acid significantly enhanced the starch fraction yields and lowered the fiber fraction yields compared to strong steeping acids such as hydrochloric acids under constant steeping conditions (initial pH value: 3; 24 h; 52 °C; 2000 ppm SO_2_) [15]. However, the steeping conditions (pH, contact time), as well as the processing steps (sieving, washing), differed from the present study, which partly explains the contrasting results.

The steeping conditions additionally affected the nutrient distribution and recovery within each fraction (see Table 1, Table A1 and Table S1). In the FF, the content and recovery of fiber-associated nutrients (TDF, AX, TPC) were greatest among all the fractions. Steeping with lactic acid at pH 2.5 resulted in the highest content of total dietary fiber (15.5–26.7%), AXs (6.63–11.3%) as well as bound phenolics (1.9–3.1 mg ferulic acid/g fraction); however, there was a slightly reduced degree of AX branching. Additionally, the increase in the pH from 2.5 to 3.5 using lactic acid resulted in a significant decline in the TDF/AX content and recovery in the FF. At pH 2.5, polysaccharides are less ionized, maintaining stable intermolecular associations. In contrast, at pH 3.5, increased ionization enhances electrostatic repulsion, converting polysaccharides into polyelectrolytes. This results in the disruption of intermolecular interactions, increasing their solubility but reducing structural integrity and recovery [22]. It was seen that the protein solubility was decreased when lowering the pH of the lactic acid solution, which adversely affected the separation of protein in the FF. As a result, the FF contained a higher protein content, while the protein recovery in the PF was slightly reduced. These findings are consistent with those previously reported by Vilcacundo et al. [23], who confirmed a strong pH-dependent protein solubility and observed a small rise in the protein solubility of selected maize protein concentrates and hydrolysates from pH 2 to 4. All the FFs showed a high starch content of up to approximately 62%, being lowest when lactic acid was applied. In addition to the steeping effects, starch separation from bran is usually influenced by the amount of washing water during sieving. In this study, the water quantity was minimized, considering also the subsequent energy-intensive processing steps (e.g., drying), which resulted in a higher starch residue in the FF. By comparing the starch recovery yields in the FF and SF (Table A1), it can be concluded that the applied steeping acid remained a key separation factor, with lactic acid specifically enhancing the separation efficiency of starch within these two fractions.

In terms of the fat content and recovery, the FF retained most of the fat present in the whole kernel, followed by the PF. As cereal lipids are found in aleurone tissue, but also mainly in the germ [24], the distribution of the germ likely contributed to its presence in both the FF and PF. The highest fat recovery was observed when using acetic acid during steeping. This treatment also resulted in the greatest amount of recovered protein in the FF. Moreover, a similar effect was noted in the protein and fat recovery yields of the SF. The application of lactic acid during steeping significantly led to the highest retention of ash in the FF, indicating potential pH dependence. This effect was most pronounced with lactic acid at pH 3.5, where the highest ash content was observed. The effect of the steeping acids at different pH levels on the yields of the wet-milled products (starch, fiber, germ, protein) has also been seen by Yang et al. [15] and is mainly influenced by the redistribution of nutrients, particularly between the FF and the PF.

Regarding the SF, this fraction yielded a similar starch content, ranging from 85.7 to 90.0%, independent of the steeping conditions. Notably, the lactic-acid-steeped fraction showed the highest purity with minimal presence from other nutrients. For the PF, the proteins were identified as the predominant component, ranging from 33.6 to 50.9% protein, with a significant increase when hydrochloric acid was used (see Table 1). Nevertheless, both the protein and fat recoveries were highest when steeped with lactic acid, particularly at pH 3.5 (see Table A1). This processing condition facilitated an increased nutrient separation from the surrounding polymer matrix, improving its overall recovery yield. The starch impurities, on the other hand, ranged from 11.9 to 27.2% and were significantly enhanced when using acetic acid. This specific steeping condition led to the least efficient starch separation in both the PF and FF, while also resulting in the lowest starch recovery in the SF. This suggested a lower dissociation of starch granules from the surrounding matrix. Similar results were reported by Yang et al. [15], who observed a significant effect of the steeping acids on the starch yields, highlighting lactic acid as the most effective acid for starch separation. Regarding ash, the PF exhibited the highest amounts of ash among the three fractions, likely due to the solubilization of minerals from the dietary fiber. This assumption is also supported by findings of Lemmens et al. [25], who suggested the improved bio-accessibility of minerals through grain steeping (pH 3.8, 50 °C, 24 h) and the occurrence of hydrolysis of the phytate structure in the bran. Additionally, the solubilization of minerals from the germ may have also contributed significantly to the ash content, as it is particularly rich in minerals [2]. The application of lactic acid (pH 2.5) potentially led to an initial increase in the solubilization of minerals during steeping and subsequent loss into the steeping water, as indicated by the lowest ash content and recovery in relation to the dietary fiber content (FF, PF). Interestingly, the AXs recovered in the PF differed from those in the FF. Higher arabinose-to-xylose (A/X) ratios were observed in all the PFs, particularly when steeped with lactic acid at pH 2.5. This observation could be attributed either to a higher branching degree or, most likely, to the enhanced release of arabinose residues induced by the applied steeping conditions. Usually, the FFs that exhibited lower arabinose contents were accompanied by an increased recovery of this monosaccharide in the respective PFs. These findings aligned with the observations of Grohmann and Bothast [26], who established that acid treatment could lead to maize fiber polysaccharide fragmentation, thereby explaining the increase in arabinose found in the PF.

In summary, steeping was found to be a critical factor influencing AX and protein, significantly affecting both their yield and key AX structural properties. Among the tested processing conditions, the acid type had the greatest impact on the recovery efficiency. At pH 2.5, the FF exhibited the highest AX content when lactic acid was used, whereas steeping with hydrochloric acid displayed the highest protein in the protein-rich fraction. Although the AX in the FF (lactic acid, pH 2.5) exhibited slightly reduced branching compared to the other treatments, it retained the highest content of bound polyphenols, which are crucial structural components for crosslinking. These polyphenols may also serve as indicators to assess the process intensity, as they are prone to cleavage under harsh processing conditions. While hydrochloric acid significantly enhanced the protein content in the PF, it caused a notable degradation of AX in the fiber fraction. In contrast, acetic acid was the least effective, yielding the least pure fractions. Increasing the pH of the steeping solution to 3.5 did not improve the overall polymer recovery.

Based on the compositional analysis and nutrient recovery, lactic acid at pH 2.5 enabled a first effective enrichment, retaining all the dietary fiber in the FF and recovering approximately 28.5% of the kernel’s protein in the PF, making this condition the most suitable for further upscaling.

### 3.2. Effect of Upscaling and PEF Pre-Treatment on Fraction Composition and Yield

Figure 2 displays the fraction recovery yields at the pilot- and lab-scale, while Table 2 (or Table S2) and Table A2 summarize the nutrient distribution and recovery yields within the three obtained fractions (FF, SF, PF), respectively. Upscaling alone and combined with PEF treatment yielded a total solid recovery of 89% and 80% from the whole kernels, respectively. Compared to lab-scale fractionation, higher handling losses were observed during the milling, sieving, and pre-processing steps. Regarding the fraction yield, the FF represented the largest fraction (untreated: 47%, treated: 43%), followed by the SF (untreated: 37%, treated: 33%). The nutritional composition and recovery yields, particularly of TDF and starch, of the FF and SF were strongly influenced by the mechanical separation of the pilot-scale processing (i.e., dynamic sieving set-up compared to static at lab-scale, sieving time, fiber-washing and sample size differences), which enhanced the residual starch in the fiber fraction. These observations were consistent with previous studies conducted by Singh et al. [27] and Rubens [28], who reported increased starch accumulation within the fiber fractions resulting from pilot-scale fractionation compared to lab-scales applying 100 g or 1 kg maize kernels.

The nutrient distribution in the FF varied significantly and closely depended on the processing scale and fractionation conditions, except for the AX and TPC content. As previously mentioned, upscaling led to a significant increase in the starch content and recovery, accompanied by a reduction in the remaining nutrients. In addition, PEF pre-treatment considerably reduced the protein, TDF, and fat content and/or recovery in the FF compared with the control (untreated). The slight yet significant reduction in the protein content in this pre-treated fraction can be attributed to increased nutrient solubilization from the cell wall matrix, which was later recovered in the PF. Li et al. [29] observed that the application of electric field strengths of up to 30 kV/cm increased the solubility, as well as the surface free sulfhydryl content and hydrophobicity, of a soy protein isolate dispersion. These effects were attributed to polarization, dissociation of protein subunits and molecular unfolding.

As mentioned before, PEF processing also led to a fat reduction in the FF and a subsequent rise in the SF and PF. A similar behavior was observed by Guderjan et al. [30], who examined the impact of PEF with field strengths of 0.6–1.3 kV/cm on the oil extraction from maize germs at different moisture contents. The variations in the total dietary fiber content between the untreated and PEF-treated FF (and the subsequent fiber distribution among the remaining fractions) can be attributed to an irreversible breakdown of the cell wall structure and a subsequent solubilization of the polysaccharides [31]. Investigations conducted by Fan et al. [13] demonstrated the efficacy of the PEF-assisted extraction of soluble dietary fiber from orange peel in enhancing the SDF yield and improving the functional properties (e.g., water-holding capacity) while reducing the molecular weight (electric field density: 6.0 kV/cm, pulse number: 30). The potential of PEF application to increase cell membrane permeability and thus facilitate the release of soluble fiber and extraction of AX was also seen by Grgić et al. [32]. Although the PEF-treated FF exhibited a lower TDF content, the AX content and structural key parameters (branching, bound phenolics) remained unaffected. This PEF-induced preservation of polyphenol stability and accessibility in cereal brans has also been observed before [33]. These findings highlighted the crucial role of PEF processing in enhancing the recovery of bioactive compounds while maintaining their structural integrity, aligning with the results of the present study.

Regarding the SF, the starch recovery was slightly reduced during upscaling. However, the additional PEF treatment increased the starch content up to about 95%. Yet this was accompanied by increased levels of fat and TDF due to solubilization effects. A similar behavior was seen in the PF, which exhibited significantly higher dietary fiber, protein and fat content and had only a small effect on the recovery yield of AXs and proteins.

### 3.3. Effect of PEF Pre-Processing on Physical Properties of Fractions

To investigate the potential structural changes induced by the PEF pre-treatment, the recovered fractions were further analyzed for their physical properties, including their pasting behavior, solvent retention capacity, solubility, and surface hydrophobicity.

#### 3.3.1. Pasting Properties

**Figure 3:**
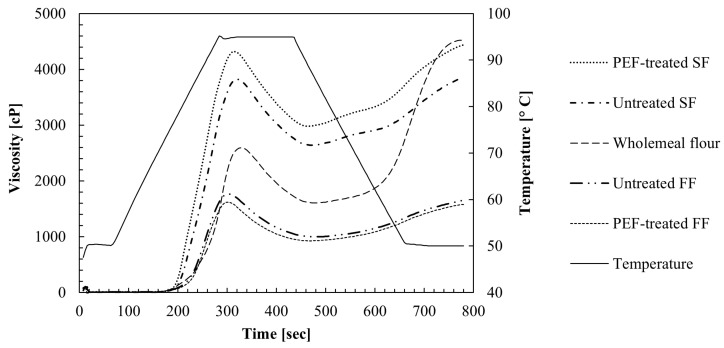
Temperature profile and pasting properties of the pilot-scale starch-rich fraction (SF), PEF-treated starch-rich fraction, fiber-rich fraction (FF) and PEF-treated fiber-rich fraction as well as the wholemeal maize flour. All the curves are based on the mean values of triplicate determinations.

The pasting profile and properties of the untreated and PEF-treated SF and FF fractions, as well as wholemeal flour, are presented in Figure 3 and Table 3, respectively. Raw data can be taken from Table S3. As expected, the SF showed significantly higher viscosities compared to the FF and the wholemeal flour, except for the latter in the final viscosity. In the SF, the observed differences in the pasting behavior between the fractions can mainly be attributed to variations in their nutritional composition. In addition to starch, dietary fiber, particularly soluble fiber, has been demonstrated to be a key factor influencing starch gelatinization and retrogradation, whereas protein and fat have contributed to a lesser extent [34]. Surprisingly, the PEF-processed fractions behaved differently in the SF and FF. While PEF enhanced the pasting profile in the SF, an opposite trend was seen in the FF. The FF only showed small compositional variations, regardless of the treatment, suggesting that the observed differences may be attributed to process-induced changes. Previous studies have reported that PEF processing can significantly affect the structural and consequently the functional properties of starch derived from various raw materials (maize, potato, tapioca, cassava). These effects are highly dependent on the applied electric field strengths and the number of pulses during treatment. High-intensity PEF treatments up to 50 kV/cm have been shown to cause depolymerization of starch granules, loss of crystallinity and consequently a reduction in the peak and breakdown viscosity [35,36], as well as gelatinization enthalpy [37], in contrast to low field strengths (<9 kV/cm), which have only shown marginal effects [38]. However, even at low electric field strengths of 1.5 kV/cm, structural changes may have been induced by the high number of pulses used in this investigation (4718 pulses). Qiu et al. [39] investigated the pulse-number-dependent impact of PEF treatment at a low field strength of 3 kV/cm on rice starch. The authors observed that an increase in the pulse number from 50 to 300 pulses resulted in a rise in the breakdown (at 95 °C after holding for 5 min) and setback (after cooling down from 95 °C to 50 °C) viscosities, a trend also observed in the PEF-treated SF of this study. While the authors also reported a significant reduction in the peak viscosity (at 95 °C), this effect was not observed in the aforementioned fraction. This discrepancy could be attributed to the slightly higher starch and fiber content as well as to the structural modifications of both polymers [35,36,37,40] in the PEF-treated SF sample, which may have affected the peak viscosity values. Although significant changes were observed in the pasting parameters, no notable change in the pasting temperature was seen following PEF application. This finding is consistent with the results of Qiu et al. [39], who reported that the pasting temperature of rice starch was not affected by PEF processing.

Interestingly, both FFs showed small differences in the peak viscosity, with the PEF-treated fraction showing a lower pasting profile despite having a higher starch (+2.6%) and a lower TDF (−5.0 %), protein (−0.2%) and fat content (−1.3%). This suggested that the PEF treatment may have also affected the structural properties of the dietary fiber within the fraction, which could have led to altered water absorption (as also later seen in Table 4) and thus to different water competition between the starch and the dietary fibers [40]. Therefore, potential alterations in the structural properties (e.g., molecular size, steric configuration) of the dietary fibers could have modified the accessibility and interaction with water within the fibers. This has been supported by Li et al. [41], who highlighted the crucial role of soluble maize fiber and starch interaction in determining the pasting characteristics. In particular, higher concentrations of soluble fiber have been shown to limit starch swelling, subsequently leading to a significant decrease in the peak, breakdown, setback and final viscosity.

#### 3.3.2. Solvent Retention Capacity

The pilot-scale FF and PF, as well as the wholemeal flour, were analyzed regarding their sucrose (SRC) and/or water retention capacity (WRC) and the results are provided in Table 4 and Table S4. While the sucrose retention capacity specifically targets the ability of pentosans to hydrate, the water retention capacity is mainly used for assessing the overall hydration of the fraction’s constituents, including pentosans, protein and damaged starch [16].

The PEF-treated fiber fraction showed a 44% higher WRC and a noticeably lower SRC compared to the untreated FF. In addition, the PEF-treated FF exhibited a small yet significant decrease in the WRC compared to the wholemeal flour while maintaining a similar SRC. The increase in the WRC also suggested structural changes in the fiber fraction resulting from the PEF processing, which led to a more porous cell wall structure. As recently reported by Karim et al. [40], the enhanced water-holding ability of dietary fibers is primarily attributed to the chemical composition of the fiber matrix, the surface properties (heterogeneity, movement flexibility, area), the degree of crystallinity and subsequently the accessibility of hydroxyl subunits. Additionally, PEF treatment is known to affect the structural and functional properties of pectin fractions (e.g., chain length, branching degree, monosaccharide composition) [42]. This fact was also confirmed by Fan et al. [13], who observed enhanced cell wall disruption in PEF-treated orange peel, leading to hydrophilic swelling of hemicelluloses due to greater molecular mobility.

Despite the similar AX content in both FFs, the SRC was significantly reduced due to the PEF treatment. Although positive correlations between the AX concentration and this property have been reported in triticale [43,44], the 5% difference in the TDF content between the FF samples may partially explain the variation in sucrose retention. Similarly, Langó et al. [44] identified relationships between the SRC, TDF (especially non-soluble dietary fiber) and free sugars. This suggests that the reduction of the sucrose retention in the PEF-treated fraction could be linked to its lower TDF content compared to the untreated fraction. To a lesser extent, the solvent retention capacities may have also been affected by PEF-induced changes to the starch, since it is the predominant component of both fractions. Han et al. [45] demonstrated that PEF-induced depolymerization of maize starch granules may result in the enhanced accessibility of water molecules.

The WRC of the PF only slightly varied due to pre-processing and was significantly lower than the FF and the wholemeal flour. The higher WRC of the PEF-PF can be mainly attributed to the higher amount of TDF. However, protein unfolding, a consequence of this pre-processing, was also observed (see Section 3.3.3), likely facilitating the interaction with water molecules. This effect usually results from the exposure of hydrophilic protein domains caused by unfolding. Similarly, Zhang et al. [46] reported that the PEF-induced unfolding of wheat gluten proteins led to an increased WRC and solubility.

#### 3.3.3. Protein Hydrophobicity and Solubility

The protein surface hydrophobicity and solubility of the PF were measured to assess the alterations in the protein structure, as shown in Table 4. Raw data can be taken from Tables S5 and S6, respectively. Significant differences in the surface hydrophobicity were observed between the PF and the wholemeal flour. The untreated PF bound 137 ± 1 µg BPB, while the treated fraction displayed a slightly lower BPB binding capacity of 128 ± < 1 µg. In contrast, the wholemeal flour exhibited low BPB binding due to the limited protein exposure within the flour’s complex matrix, which restricts BPB access to protein sites.

The reduction of the protein surface hydrophobicity in the PEF-treated PF may be attributed to the PEF-induced modifications to the protein’s secondary structure, resulting in the exposure of internal hydrophilic amino acids on the polymer surface. Similar effects were reported by Qiu et al. [39], who observed significant changes in the secondary structure (β-sheets, α-helices), as well as in the carbonyl and amino groups, after PEF treatment of rice flours. However, the extent of the structural alterations strongly depends on the applied parameters, such as the field strength, pulse duration or frequency, as well as the type of protein [12].

Regarding the protein solubility, no differences were seen in the fractions regardless of their pre-treatment. A slight, though not statistically significant, increase in solubility was measured in the treated PF compared to the untreated fraction. As expected, the wholemeal flour showed significantly lower protein solubility, probably due to the wet fractionation, which predominantly recovers soluble proteins. Insoluble proteins tend to remain in the bran fraction, as these are not easily solubilized from the tightly packed flour matrix [47].

Based on these results, the protein alterations due to PEF treatment were minimal, with only minor modifications to the surface hydrophobicity observed, suggesting that the overall protein structure was largely preserved during PEF-processing.

## 4. Conclusions

This study examined the effect of different wet fractionation conditions and the application of PEF pre-treatment on the efficient and gentle recovery of AXs and proteins. The findings highlighted the importance of the steeping parameters, which significantly affected the nutritional distribution, yield and structural properties of the fractions, particularly of AX. Steeping with lactic acid (pH 2.5) proved to be the most efficient condition for recovering TDF and AX, which were predominantly retained in the FF. Additionally, this treatment had the least influence on the bound polyphenol content in the FF, preserving the esterified AX structures the most.

The application of a PEF pre-treatment prior to fractionation enhanced the release of proteins and fat from the FF matrix, subsequently enriching the PF. However, this processing slightly reduced the purity of the remaining fractions (SF and PF), as a small proportion of dietary fiber was also solubilized along with these. Physical characterization of the fractions revealed PEF-induced structural modifications, particularly in the FF, enhancing the water-holding and pasting behaviors of the fiber while showing only small effects on the protein structure in the PF.

Overall, this research highlighted the importance of optimizing traditional wet fractionation processes to achieve a balance between the efficiency and the gentle recovery of fractions, as the structural integrity of the polymers within the fractions can be significantly altered. It also provided the first insights into the potential of PEF as a tool for enhancing fractionation efficiency and improving resource utilization. Future research will focus on exploring how the structural properties of proteins and AXs in the recovered fractions influence crosslinking reactions, which could present a novel approach to stabilizing food systems.

## Figures and Tables

**Table 1:** Effect of the steeping acid (acetic acid, AA; hydrochloric acid, HA; lactic acid, LA) and pH on the nutritional composition of lab-scale maize fractions.

Steeping Conditions	Protein	Fat	Total Starch	Ash	Total Dietary Fiber	Arabinoxylan ^1^	AX Ratio	Bound Phenolic Acids ^2^	Conjugated Phenolic Acids ^2^	Free Phenolic Acids ^2^
	*Fiber-rich maize fractions—FF [g/100 g fraction, d. b. ^3^]*									
**AA, pH 2.5**	7.9 ± <0.1 ^c^	5.8 ± 0.1 ^a^	62.1 ± 0.9 ^c^	0.50 ± 0.01 ^a^	15.5 ± 0.6 ^a^	6.85 ± 0.09 ^a^	1.21 ± 0.05 ^b^	2.3 ± 0.2 ^b^	0.376 ± 0.024 ^b^	0.102 ± 0.028 ^a^
**HA, pH 2.5**	7.2 ± <0.1 ^a^	6.3 ± 0.1 ^b^	58.1 ± 0.6 ^b^	0.59 ± 0.06 ^b^	17.3 ± 0.7 ^b^	6.63 ± 0.35 ^a^	1.05 ± 0.12 ^a,b^	1.9 ± <0.1 ^a^	0.156 ± 0.039 ^a^	0.181 ± 0.025 ^b^
**LA, pH 2.5**	8.6 ± 0.1 ^d^	7.32 ± 0.09 ^c^	54.6 ± 0.4 ^a^	0.67 ± 0.01 ^c^	26.7 ± 0.3 ^d^	11.32 ± 0.55 ^c^	0.88 ± 0.06 ^a^	3.1 ± 0.2 ^c^	0.320 ± 0.066 ^b^	0.118 ± 0.010 ^a^
**LA, pH 3.5**	7.7 ± <0.1 ^b^	7.32 ± 0.08 ^c^	54.3 ± 0.7 ^a^	0.72 ± 0.01 ^d^	20.7 ± 0.8 ^c^	9.95 ± 0.43 ^b^	1.02 ± 0.11 ^a^	2.4 ± 0.1 ^b^	0.378 ± 0.075 ^b^	0.168 ± 0.013 ^b^
	*Starch-rich maize fractions—SF [g/100 g fraction, d. b.]*									
**AA, pH 2.5**	1.2 ± 0.1 ^c^	0.101 ± 0.003 ^d^	87.5 ± 0.8 ^a^	0.09 ± <0.01 ^a^	n. d. ^4^	- ^5^	-	-	-	-
**HA, pH 2.5**	0.41 ± 0.01 ^a^	0.007 ± 0.001 ^a^	85.7 ± 2.0 ^a^	0.15 ± <0.01 ^a^	n. d.	-	-	-	-	-
**LA, pH 2.5**	0.59 ± 0.11 ^b^	0.060 ± 0.003 ^c^	90.0 ± 0.8 ^b^	0.13 ± 0.01 ^a^	n. d.	-	-	-	-	-
**LA, pH 3.5**	0.46 ± 0.02 ^a,b^	0.055 ± 0.010 ^b^	87.7 ± 1.2 ^a,b^	0.14 ± <0.01 ^a^	n. d.	-	-	-	-	-
	*Protein-rich maize fractions—PF [g/100 g fraction, d. b.]*									
**AA, pH 2.5**	33.6 ± 1.9 ^a^	5.0 ± 0.1 ^b^	27.2 ± 0.5 ^c^	7.3 ± 0.1 ^a^	8.4 ± 0.2 ^a^	3.37 ± 0.39 ^b^	1.67 ± 0.22 ^a^	-	-	-
**HA, pH 2.5**	50.9 ± 0.8 ^d^	7.1 ± 0.1 ^c^	12.2 ± <0.1 ^a^	10.9 ± 0.4 ^d^	10.0 ± 1.1 ^b^	3.25 ± 0.33 ^b^	1.74 ± 0.21 ^a^	-	-	-
**LA, pH 2.5**	43.0 ± 0.4 ^b^	4.7 ± 0.1 ^a^	20.4 ± <0.1 ^b^	8.3 ± 0.2 ^b^	10.7 ± 1.3 ^b^	3.62 ± 0.12 ^b^	2.94 ± 0.22 ^b^	-	-	-
**LA, pH 3.5**	47.8 ± 0.3 ^c^	8.7 ± <0.1 ^d^	11.9 ± 0.5 ^a^	10.4 ± <0.1 ^c^	9.5 ± 0.8 ^a,b^	2.70 ± 0.05 ^a^	1.46 ± 0.20 ^a^	-	-	-
	*Wholemeal fraction—WF [g/100 g wholemeal flour, d. b.]*									
**Wholemeal flour**	6.77 ± 0.10	4.18 ± 0.45	65.2 ± 1.4	1.32 ± <0.01	10.0 ± 0.3	4.07 ± 0.12	1.03 ± 0.01	3.71 ± 0.15	0.31 ± 0.02	0.24 ± 0.04

Results are presented as the mean values of triplicate determinations ± standard deviation. Superscript letters denote significant differences (*p*-value ≤ 0.05) within each nutrient in each fraction. ^1^ AX was calculated as the sum of arabinose and xylose; ^2^ expressed as mg ferulic acid/g dry fraction; ^3^ d. b.: dry base; ^4^ n. d.: not detected; ^5^ not measured.

**Table 2:** Effect of upscaling (pilot scale, PS) and pulsed electric field (PEF) pre-treatment on thenutritional composition of fractions steeped with lactic acid (LA) at a pH of 2.5.

Processing Conditions	Protein	Fat	Total Starch	Ash	Total Dietary Fiber	Arabinoxylan ^1^	AX Ratio	Bound Phenolic Acids ^2^	Conjugated Phenolic Acids ^2^	Free Phenolic Acids ^2^
	*Fiber-rich maize fractions—FF [g/100 g fraction, d. b. ^3^]*									
Lab-scale	8.57 ± 0.07 ^c^	7.3 ± 0.1 ^c^	54.6 ± 0.4 ^a^	0.67 ± 0.01 ^c^	26.7 ± 0.3 ^c^	11.32 ± 0.55 ^b^	0.88 ± 0.06 ^b^	3.1 ± 0.2 ^b^	0.32 ± 0.07 ^a^	0.118 ± 0.010 ^a^
PS, untreated	6.74 ± 0.07 ^b^	4.4 ± 0.2 ^b^	57.2 ± 1.1 ^b^	0.55 ± 0.01 ^b^	16.0 ± 0.7 ^b^	5.17 ± 0.14 ^a^	0.72 ± <0.01 ^a^	1.8 ± 0.2 ^a^	0.54 ± 0.24 ^a,b^	0.121 ± 0.026 ^a^
PS + PEF	6.55 ± 0.05 ^a^	3.1 ± <0.1 ^a^	59.8 ± 1.4 ^c^	0.44 ± <0.01 ^a^	11.0 ± 0.2 ^a^	5.22 ± 0.09 ^a^	0.71 ± 0.01 ^a^	2.3 ± 0.4 ^a^	0.84 ± 0.13 ^b^	0.104 ± 0.016 ^a^
	*Starch-rich maize fractions—SF [g/100 g fraction, d. b.]*									
Lab-scale	0.59 ± 0.11 ^a^	0.06 ± <0.01 ^a^	90.0 ± 0.8 ^a^	0.130 ± 0.006 ^b^	n. d. ^4^	n. d.	- ^5^	-	-	-
PS, untreated	0.52 ± 0.06 ^a^	0.05 ± <0.01 ^a^	87.9 ± 1.4 ^a^	0.096 ± 0.001 ^a^	1.3 ± 0.1 ^a^	n. d.	-	-	-	-
PS + PEF	0.63 ± 0.02 ^a^	2.00 ± 0.11 ^b^	95.3 ± 1.2 ^b^	0.137 ± 0.007 ^b^	2.3 ± 0.4 ^b^	n. d.	-	-	-	-
	*Protein-rich maize fractions –PF [g/100 g fraction, d. b.]*									
Lab-scale	43.0 ± 0.4 ^c^	4.7 ± 0.1 ^b^	20.4 ± <0.1 ^b^	8.3 ± 0.2 ^c^	10.7 ± 1.4 ^b^	3.62 ± 0.12 ^c^	2.94 ± 0.22 ^b^	-	-	-
PS, untreated	39.8 ± 0.2 ^a^	2.0 ± 0.1 ^a^	21.1 ± 0.7 ^b^	7.3 ± 0.1 ^b^	7.7 ± 1.3 ^a^	2.42 ± 0.04 ^a^	1.21 ± 0.02 ^a^	-	-	-
PS + PEF	41.9 ± <0.1 ^b^	17 ± 1 ^c^	17.1 ± 1.0 ^a^	6.8 ± 0.3 ^a^	11.3 ± 0.3 ^b^	2.65 ± 0.05 ^b^	1.17 ± 0.04 ^a^	-	-	-
	*Wholemeal fraction—WF [g/100 g wholemeal flour, d. b.]*									
**Wholemeal flour**	6.77 ± 0.10	4.2 ± 0.5	65.2 ± 1.4	1.3 ± <0.1	10.0 ± 0.3	4.07 ± 0.12	1.03 ± 0.01	3.71 ± 0.15	0.31 ± 0.02	0.24 ± 0.04

Results are presented as the mean values of triplicate determinations ± standard deviation. Superscript letters denote significant differences (*p*-value ≤ 0.05) within each nutrient in each fraction. ^1^ AX was calculated as the sum of arabinose and xylose; ^2^ expressed as mg ferulic acid/g dry fraction; ^3^ d. b.: dry base; ^4^ n. d.: not detected; ^5^ not measured.

**Table 3:** Effect of PEF treatment on the pasting properties of the starch-rich (SF) and fiber-rich fractions (FF) produced at pilot scale compared to native wholemeal maize flour.

Maize Fraction	Peak Viscosity [cP]	Trough Viscosity [cP]	Final Viscosity [cP]	Breakdown Viscosity [cP]	Setback Viscosity [cP]	Pasting Temperature [°C]
**Untreated SF**	3826 ± 151 ^d^	3027 ± 647 ^c^	3846 ± 131 ^b^	1188 ± 20 ^c^	1207 ± 42 ^b^	76.1 ± <0.5 ^a^
**PEF-treated SF**	4323± 50 ^e^	2976 ± 59 ^c^	4365 ± 50 ^c^	1347 ± 94 ^d^	1457 ± 11 ^c^	75.4 ± 0.5 ^a^
**Untreated FF**	1776 ± 44 ^b^	995 ± 8 ^a^	1644 ± 95 ^a^	781 ± 37 ^a^	649 ± 88 ^a^	78.5 ± 1.9 ^b^
**PEF-treated FF**	1625 ± 18 ^a^	925 ± 14 ^a^	1578 ± 17 ^a^	700 ± 10 ^a^	653 ± 6 ^a^	79.9 ± <0.1 ^b^
**Wholemeal flour**	2594 ± 4 ^c^	1606 ± 6 ^b^	4519 ± 19 ^c^	989 ± 10 ^b^	2914 ± 18 ^d^	75.8 ± <0.1 ^a^

Results are presented as the mean values of triplicate determinations ± standard deviation. Superscript letters denote significant differences (*p*-value ≤ 0.05) within each column.

**Table 4:** Selected physical properties (solvent retention capacity, protein hydrophobicity, protein solubility) of the untreated and PEF-treated fiber-rich (FF) and protein-rich fractions (PF) compared to the wholemeal maize flour.

Fraction		Water-Holding Capacity [%]	Sucrose Retention Capacity [%]	Surface Hydrophobicity [µg BPB ^1^]	Protein Solubility [%]
**Wholemeal flour**		129 ± 2 ^d^	111 ± 1 ^a^	34.6 ± 6.0 ^a^	14.0 ± 1.3 ^a^
**FF**	**Untreated**	89.3 ± 0.8 ^c^	157 ± 4 ^b^	- ^2^	-
	**PEF-treated**	133 ± 1 ^e^	108 ± 2 ^a^	-	-
**PF**	**Untreated**	29.0 ± 0.9 ^a^	-	137 ± 1 ^c^	51.2 ± 0.6 ^b^
	**PEF-treated**	39.5 ± 1.2 ^b^	-	128 ± <1 ^b^	52.3 ± <1.0 ^b^

Results are presented as the mean values of triplicate determinations ± standard deviation. Superscript letters denote significant differences (*p*-value ≤ 0.05) within each column; ^1^ bromophenol blue; ^2^ not determined.

## Data Availability

All data generated or analyzed during this study are included in this published article.

## Supplementary Materials

The following supporting information can be downloaded at https://www.mdpi.com/article/10.3390/foods14050760/s1, Table S1: Effect of the steeping acid (acetic acid, AA; hydrochloric acid, HA; lactic acid, LA) and pH on fraction yield and on the nutritional composition of lab-scale maize fractions (raw data); Table S2: Effect of upscaling (pilot scale, PS) and pulsed electric field (PEF) pre-treatment on fraction yield and nutritional composition of fractions steeped with lactic acid (LA) at a pH of 2.5 (raw data); Table S3: Pasting properties of the pilot-scale starch-rich fraction (SF), PEF-treated starch-rich fraction, fiber-rich fraction (FF), PEF-treated fiber-rich fraction and wholemeal maize flour (raw data); Table S4: Solvent retention capacity of the untreated and PEF-treated fiber-rich (FF), protein-rich fractions (PF) and wholemeal maize flour. Table S5: Protein hydrophobicity of the untreated and PEF-treated fiber-rich (FF), protein-rich fractions (PF) and the wholemeal maize flour (raw data).Table S6: Protein solubility of the untreated and PEF-treated fiber-rich (FF), protein-rich fractions (PF) and wholemeal maize flour (raw data).

## Appendix A

**Table A1:** Effect of the steeping acids (acetic acid, AA; hydrochloric acid, HA; lactic acid, LA) and pH on the nutrient recovery yield [%] of lab-scale maize fractions.

Steeping Conditions	Protein	Fat	Total Starch	Ash	Total Dietary Fiber	Arabinoxylan	Bound Phenolic Acids	Conjugated Phenolic Acids	Free Phenolic Acids
*Fiber-rich maize fractions—FF*									
AA, pH 2.5	60.12 ± 0.60 ^c^	72.01 ± 8.00 ^b^	49.23 ± 1.10 ^c^	19.77 ± 0.46 ^a^	80.00 ± 2.90 ^a^	86.98 ± 1.23 ^b^	32.35 ± 2.43 ^b^	61.29 ± 4.55 ^c^	22.08 ± 5.83 ^a^
HA, pH 2.5	49.63 ± 0.50 ^b^	70.10 ± 7.60 ^a^	41.56 ± 0.95 ^b^	20.83 ± 0.23 ^b,c^	80.30 ± 3.10 ^a,b^	75.92 ± 3.93 ^a^	23.18 ± 0.54 ^a^	22.58 ± 6.45 ^a^	35.00 ± 5.00 ^b^
LA, pH 2.5	50.07 ± 0.55 ^b^	69.38 ± 7.60 ^a^	33.12 ± 0.55 ^a^	20.23 ± 0.23 ^b^	105.60 ± 1.00 ^a,b^	110.07 ± 5.50 ^d^	32.88 ± 2.43 ^b^	41.94 ± 9.68 ^b^	19.58 ± 1.67 ^a^
LA, pH 3.5	44.30 ± 0.45 ^a^	68.18 ± 7.30 ^a^	32.37 ± 0.95 ^a^	21.36 ± 0.23 ^c^	80.40 ± 3.00 ^a^	95.33 ± 4.25 ^c^	24.80 ± 1.35 ^a^	48.39 ± 9.68 ^b^	27.08 ± 2.08 ^b^
*Starch-rich maize fractions—SF*									
AA, pH 2.5	6.06 ± 0.59 ^c^	0.84 ± 0.02 ^c^	46.32 ± 1.00 ^a^	9.39 ± 6.14 ^a^	n. d. ^1^	- ^2^	-	-	-
HA, pH 2.5	2.51 ± 0.15 ^a^	0.07 ± 0.01 ^a^	53.38 ± 1.23 ^b^	13.64 ± 7.88 ^a^	n. d.	-	-	-	-
LA, pH 2.5	4.14 ± 0.74 ^b^	0.69 ± 0.02 ^c^	65.79 ± 1.00 ^c^	4.70 ± 0.23 ^a^	n. d.	-	-	-	-
LA, pH 3.5	3.10 ± 0.15 ^a^	0.62 ± 0.10 ^b^	62.74 ± 1.00 ^c^	4.92 ± 0.23 ^a^	n. d.	-	-	-	-
*Protein-rich maize fractions—PF*									
AA, pH 2.5	21.71 ± 1.18 ^a^	5.26 ± 0.24 ^a^	1.83 ± 0.04 ^d^	24.24 ± 0.76 ^a^	3.80 ± 0.20 ^a^	3.64 ± 0.42 ^b^	-	-	-
HA, pH 2.5	27.61 ± 0.44 ^b^	6.22 ± 0.24 ^b^	0.69 ± 0.02 ^a^	30.30 ± 0.76 ^c^	3.70 ± 0.40 ^a^	2.92 ± 0.29 ^a^	-	-	-
LA, pH 2.5	28.51 ± 0.30 ^b^	5.02 ± 0.24 ^a^	1.41 ± 0.02 ^c^	28.03 ± 0.76 ^b^	4.80 ± 0.60 ^b^	4.00 ± 0.12 ^b^	-	-	-
LA, pH 3.5	31.61 ± 0.15 ^c^	9.33 ± 0.24 ^c^	0.81 ± 0.02 ^b^	34.85 ± 0.76 ^d^	4.30 ± 0.40 ^a, b^	2.97 ± 0.05 ^a^	-	-	-

Results are presented as the mean values of triplicate determinations ± standard deviation. Superscript letters denote significant differences (*p*-value ≤ 0.05) within each nutrient in each fraction. ^1^ n. d.: not detected; ^2^ not measured.

**Table A2:** Effect of the upscaling (pilot-scale, PS) and pulsed electric field (PEF) pre-treatment on the nutrient recovery yield [%] of fractions steeped with LA (LA) at a pH of 2.5.

Processing Conditions	Protein	Fat	Total Starch	Ash	Total Dietary Fiber	Arabinoxylan	Bound Phenolic Acids	Conjugated Phenolic Acids	Free Phenolic Acids
*Fiber-rich maize fractions—FF*									
Lab-scale	50.07 ± 0.44 ^c^	69.38 ± 1.15 ^c^	33.12 ± 0.15 ^a^	20.23 ± 0.23 ^c^	105.60 ± 1.00 ^c^	110.07 ± 5.50 ^c^	32.88 ± 2.43 ^b^	41.94 ± 9.68 ^a^	19.58 ± 1.67 ^a^
PS, untreated	46.82 ± 0.59 ^b^	49.04 ± 2.15 ^b^	41.27 ± 0.77 ^c^	19.47 ± 0.53 ^b^	75.00 ± 3.00 ^b^	59.95 ± 1.72 ^b^	22.38 ± 1.89 ^a^	80.65 ± 35.48 ^a,b^	23.75 ± 5.00 ^a^
PS + PEF	41.80 ± 0.30 ^a^	31.82 ± 0.24 ^a^	39.57 ± 0.92 ^b^	14.39 ± 0.08 ^a^	48.00 ± 1.00 ^a^	55.28 ± 0.98 ^a^	26.42 ± 4.58 ^a^	116.13 ± 19.35 ^b^	18.75 ± 2.92 ^a^
*Starch-rich maize fractions—SF*									
Lab-scale	4.14 ± 0.74 ^b^	0.69 ± 0.02 ^a^	65.79 ± 1.00 ^b^	4.70 ± 0.23 ^c^	n. d. ^1^	n. d.	- ^2^	-	-
PS, untreated	2.81 ± 0.30 ^a^	0.48 ± 0.24 ^a^	50.00 ± 0.77 ^a^	2.73 ± 0.08 ^a^	5.00 ± 0.30 ^a^	n. d.	-	-	-
PS + PEF	3.10 ± 0.15 ^a^	15.79 ± 0.95 ^b^	48.16 ± 0.62 ^a^	3.41 ± 0.15 ^b^	7.50 ± 1.30 ^b^	n. d.	-	-	-
*Protein-rich maize fractions—PF*									
Lab-scale	28.51 ± 0.30 ^c^	5.02 ± 0.24 ^b^	1.41 ± 0.02 ^b^	28.03 ± 0.76 ^c^	4.75 ± 0.72 ^b^	4.00 ± 0.12 ^b^	-	-	-
PS, untreated	26.58 ± 0.15 ^b^	2.15 ± 0.24 ^a^	1.46 ± 0.05 ^b^	25.00 ± 0.76 ^b^	3.50 ± 0.58 ^a^	2.68 ± 0.05 ^a^	-	-	-
PS + PEF	24.51 ± 0.15 ^a^	15.79 ± 0.48 ^c^	1.03 ± 0.06 ^a^	20.45 ± 0.76 ^a^	4.46 ± 0.13 ^b^	2.55 ± 0.05 ^a^	-	-	-

Results are presented as the mean values of triplicate determinations ± standard deviation. Superscript letters denote significant differences (*p*-value ≤ 0.05) within each nutrient in each fraction. ^1^ n. d.: not detected; ^2^ not measured.
